# High Prevalence of Hepatitis E Virus in Swedish Moose – A Phylogenetic Characterization and Comparison of the Virus from Different Regions

**DOI:** 10.1371/journal.pone.0122102

**Published:** 2015-04-23

**Authors:** Jay Lin, Marie Karlsson, Ann-Sophie Olofson, Sándor Belák, Jonas Malmsten, Anne-Marie Dalin, Frederik Widén, Heléne Norder

**Affiliations:** 1 Department of Virology, Immunobiology and Parasitology (VIP), National Veterinary Institute (SVA), Uppsala, Sweden; 2 Swedish University of Agricultural Sciences (SLU), Department of Biomedical Science and Veterinary Public Health, Uppsala, Sweden; 3 Department of Infectious Diseases/Section of Clinical Virology, Institute of Biomedicine, University of Gothenburg, Gothenburg, Sweden; 4 Department of Pathology and Wildlife Diseases, National Veterinary Institute (SVA), Uppsala, Sweden; 5 Division of Reproduction, Department of Clinical Sciences, Swedish University of Agricultural Sciences (SLU), Uppsala, Sweden; Virginia Polytechnic Institute and State University, UNITED STATES

## Abstract

**Background:**

Hepatitis E virus (HEV) infects a range of species, including humans, pigs, wild boars and deer. Zoonotic transmission may contribute to the high HEV seroprevalence in the human population of many countries. A novel divergent HEV from moose (*Alces alces*) in Sweden was recently identified by partial genome sequencing. Since only one strain was found, its classification within the HEV family, prevalence in moose and zoonotic potential was unclear. We therefore investigated samples from 231 moose in seven Swedish counties for HEV, and sequenced a near complete moose HEV genome. Phylogenetic analysis to classify this virus within the family *Hepeviridae* and to explore potential host specific determinants was performed.

**Methods and Findings:**

The HEV prevalence of moose was determined by PCR (marker for active infection) and serological assays (marker of past infection) of sera and 51 fecal samples from 231 Swedish moose. Markers of active and past infection were found in 67 (29%) animals, while 34 (15%) were positive for HEV RNA, 43 (19%) were seropositive for anti-HEV antibodies, and 10 (4%) had both markers. The number of young individuals positive for HEV RNA was larger than for older individuals, and the number of anti-HEV antibody positive individuals increased with age. The high throughput sequenced moose HEV genome was 35-60% identical to existing HEVs. Partial ORF1 sequences from 13 moose strains showed high similarity among them, forming a distinct monophyletic clade with a common ancestor to HEV genotype 1-6 group, which includes members known for zoonotic transmission.

**Conclusions:**

This study demonstrates a high frequency of HEV in moose in Sweden, with markers of current and past infection demonstrated in 30% of the animals. Moose is thus an important animal reservoir of HEV. The phylogenetic relationship demonstrated that the moose HEV belonged to the genotype 1-6 group, which includes strains that also infect humans, and therefore may signify a potential for zoonotic transmission of this HEV.

## Introduction

Hepatitis E is the most common cause of acute viral hepatitis [1]. The disease is generally self-limiting, but immunocompromised patients such as solid organ transplanted or HIV-infected individuals, are at risk to become chronically infected, with rapid development of fibrosis and cirrhosis [1,2]. The infectious agent, hepatitis E virus (HEV), is the only member of the family *Hepeviridae* in the genus *Hepevirus*, recently proposed to be renamed to *Orthohepevirus* [3] or divided into five new genera *Orthohepevirus*, *Rocahepevirus*, *Chiropteranhepevirus*, *Avihepevirus and Pishihepevirus* [4]. It is a small, non-enveloped virus with a single-stranded, positive-sense RNA genome ranging from 6.6–7.3 kb depending on the strain. The genome encodes for three open reading frames (ORF1-3), with ORF1 and 3 encoding for non-structural proteins, and ORF2 for the capsid protein [5]. Hepeviruses that infect humans are proposed to be classified into the species *Orthohepevirus A*, which consists of at least six genotypes (Gt1-6) [3], or into the proposed genus *Orthohepevirus*, with at least six species HEV1-6 [4]. At least those strains belonging to Gt1-4 appear to share the same serotype, i.e. infection with one genotype infers immunity against the other types [5]. Gt1 and Gt2 infect only humans and are associated with large waterborne outbreaks in endemic countries in Asia and Africa, with high mortality rates in infected pregnant women and young infants [1,2,6–8]. Gt3 and Gt4 are zoonotic and are infecting animals and cause of sporadic human infections [8]. Gt3 is found worldwide especially in Europe, the United States, and Japan, while Gt4 is prevalent in Asia, but now appears to be circulating also in Europe including Scandinavia [8–11]. Wild boar and domestic swine are frequently infected with HEV and are the main reservoirs for Gt3 and Gt4 [8,12]. Highly divergent hepeviruses have been detected in a wide range of animals including rats, ferrets, bats, poultry, and cutthroat trout [13–17]. The list is expected to expand as more species are investigated, hence an updated unified consensus HEV classification system is needed [3,4].

The transmission pathways for Gt3 and Gt4 are somewhat obscure. Zoonotic transmission through ingestion of undercooked meat of HEV-infected reservoirs have been documented [18–21], and it has been shown to be transmitted through organ transplantation, blood or blood products [8]. HEV RNA has been identified in one out of 4,500 German and one out of 8,000 Swedish blood donations [22]. However, other as yet unidentified transmission routes may also exist, since the seroprevalance against HEV is high in most European countries and varies from 9% to 53%, depending on the assay used for anti-HEV antibody detection, as well as other factors [1,23,24]. Working and coming in close contact with animals (e.g. veterinary, slaughter house and forestry personnel) appears to be a risk factor for HEV transmissions, as reflected in increased HEV seroprevalence compared to the general public [8,25]. Despite the high seroprevalence, few clinical cases are reported; this may be due to either a high rate of sub-clinical infections or infections that are undiagnosed or misdiagnosed [1]. Zoonotic transmissions may contribute to the high seroprevalence, since at least 8% of Swedish wild boars (all ages) and 30% of domestic swine (age group 2–4 months) are infected with Gt3 at a given time point [12]. Deer (family *Cervidae*) have been found to be infected with Gt3 and Gt4 in Europe and Asia [18,26], and have also been linked to human HEV infections [18,20,21]. The family *Cervidae* consists of 23 genera containing 47 species including three subfamilies: *Capriolinae*, *Cervinae* and *Hydropotinae* [27]. The largest deer, moose (*Alces alces*), is a member of the *Capriolinae* and is common in Scandinavia as well as other countries around the Baltic Sea, in North America, and in northern Asia. This large deer species has previously not been studied regarding HEV infection, but a Swedish moose was recently shown to be infected with an HEV-like virus, which was subsequently characterized by partial sequencing [28]. The HEV prevalence in moose and its possible zoonotic potential was investigated in this study by analyzing samples collected from moose in seven Swedish counties and by sequencing and analyzing a near-complete HEV genome from an HEV-infected moose.

## Materials and Methods

### Sample origin and preparation

**Fig 1:**
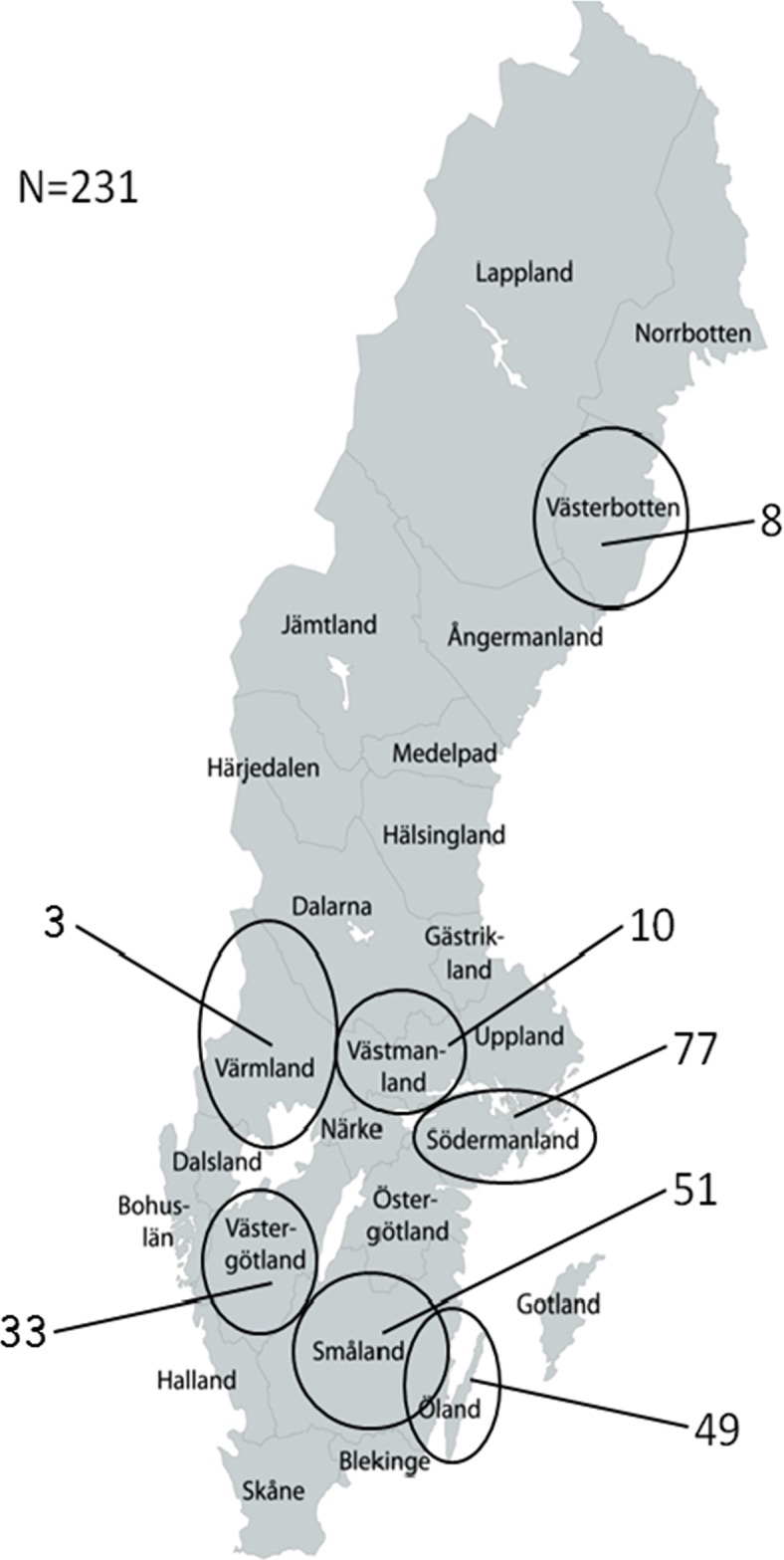
Map illustrating number of moose samples collected from Swedish counties in this study: The island of Öland, Småland, Västergötland, Södermanland, Västmanland Värmland, and Västerbotten.

Samples from 231 moose were used for detection of HEV RNA and anti-HEV antibodies. Out of these, 57 serum and 51 fecal samples from 57 moose were sent in by hunters, while serum samples previously collected from 173 moose as part of a study on *Anaplasma* infections were also provided with the assistance of hunters [29]. An additional moose liver sample positive for HEV [28] was also included in this study. The moose samples were obtained from seven Swedish counties: the Island of Öland, Småland, Västergötland, Södermanland, Västmanland, Värmland and Västerbotten (Fig 1). Most samples were collected during September to November 2012 and 2013. For most animals, pathological examinations were performed in the field by a trained wildlife pathologist who, after being contacted by hunters, travelled to the recently harvested moose carcass to collect adequate samples for the parallel *Anaplasma* study [29]. The carcass and its internal organs were thus macroscopically inspected for lesions. Moose were sampled in accordance with EU legislations on animal research (permit numbers C194/7 and C124/11 issued by the ethical committee on animal research in Uppsala, Sweden). Additional ethical and other approvals were not needed, since the moose were killed with a hunting rifle by certified hunters during permitted hunting season according to the Swedish legislation. The hunters primary hunted moose for private consumption, but voluntarily shared samples for this study. All efforts were made to minimize animal suffering and this project did not involve endangered or protected species.

### HEV-specific antibody detection by ELISA

For detection of total HEV-specific antibodies, moose sera were tested by double antigen sandwich ELISA (HEV Ab EIA, Axiom Diagnostics, Worms, Germany), performed according to the manufacturer’s instructions and the optical density (OD) was measured at 450 nm with Multiscan EX (ThermoLabsystems, Vantaa, Finland).

### RNA isolation and cDNA synthesis

Liver and fecal samples were homogenized in grinding tubes as described previously [12,28] and used for RNA isolation or frozen at -20 or -70°C for longterm storage. RNA in serum and feces was extracted with QIAamp Viral RNA mini kit (Qiagen, Germany) according to manufacturers’ instructions. For RNA extraction from liver, the Qiagen RNEasy Mini kit was used according to the manufacturer’s instructions and cDNA synthesis was performed as described previously [28]. cDNA from liver selected for MiSeq sequencing was converted to double-stranded DNA (dsDNA) by adding 0.5μl Klenow Fragment DNA polymerase (3’→5’exonuclase-negative; New England Biolabs, USA) in for 1 hour at 37°C, followed by 10 min inactivation at 75°C.

### HEV RNA detection by qPCR and PCR typing assay

Detection of HEV RNA in the moose samples was performed with a one step TaqMan qPCR assay targeting the ORF2/3 overlapping region using the Qiagen One step RT-PCR kit. A plasmid with 2.1 kb sequence homologous to partial moose HEV genome [28], including the targeted region, was used as positive control. The 12.5 μl PCR-mix contained 3 μl of purified RNA, 600 nM each of primers HEV F8, HEV R8, and FAM-based probe P8 (Table 1), 1X PCR buffer, and 1X enzyme mix. Samples were analyzed on a Rotor-Gene 3000 instrument (Corbett Research, UK) with the following settings: 50°C for 30 min, 94°C for 15 min, cycled 55 times between 94°C 15s and 60°C 60s.

**Table 1:** Moose HEV PCR primers and probe used in the study.

	Primer name	Primer type	Sequence
**qPCR**			
	HEVF8	Forward	AGGTGGTGGTTGGGGCCCT
	HEVR8	Forward	TGGCGAATGGGTTTGAGGGG
	HEVP8	Forward	CGCCTCGACTCGCAGCCATTTGC
	(FAM probe)		
**Primers used for HEV typing**			
**First PCR**			
Pool 1	ISP-4232A	Forward	GCATTTCRGCYTGGAGYAAGAC
Pool 1	ISP-4232B	Forward	GTATATCGGCCTGGAGYAAGAC
Pool 1	ISP-4232E	Forward	GYATATCGGCTTGGAGCAAGAC
Pool 2	EAP-4576F	Reverse	AGYGTRCCRGGCTCACCRGA
Pool 2	EAP-4576E	Reverse	AAYGTACCGGGCTCACCRGA
**Semi-nested PCR**			
Pool 1	ISP-4232A	Forward	GCATTTCRGCYTGGAGYAAGAC
Pool 1	ISP-4232B	Forward	GTATATCGGCCTGGAGYAAGAC
Pool 1	ISP-4232E	Forward	GYATATCGGCTTGGAGCAAGAC
Pool 3	IAP-4561E	Reverse	TCACCAGARTGTTTYTTCCATCG
Pool 3	IAP-4561F	Reverse	TCTCCAGAGTGCTTTTTCCAGCA
Pool 3	IAP-4561M	Reverse	TCACCAGAATGTTTTTTCCAACTG

A 350 nt-long region of the RNA dependent RNA-polymerase (RdRp) in ORF1, commonly used for HEV typing, was amplified according to a published seminested PCR protocol [30] with the following modifications: In the first PCR, the total reaction volume was 15 μl containing 1X buffer, 0.2 μM dNTP, 1 mM MgCl_2_, 0.6 U of platinum Taq polymerase and three pooled modified forward primers (Pool 1: ISP-4232A, ISP-4232B and ISP-4232E) and two pooled modified reverse primers (Pool 2: EAP-4576E and EAP-4576F) with a total concentration of 0.2 μM for each pool (Table 1). The semi-nested PCR contained the same reagents as the first PCR except that the reverse primer was replaced with three modified pooled primers (Pool 3: IAP-4561E, IAP-4561F and IAP-4561M). Both PCR reactions started with 94°C for 3 min, followed by cycling for 40 times between 94°C for 40s, 56°C for 30s and 72°C for 65s and finally 72°C for 10 min. A double nested-PCR with the same primers as in the second PCR was used for two purified PCR products with low amplicon concentration. PCR products were visualized by 0.8% agarose gel electrophoresis, excised, purified, and Sanger-sequenced as described previously [28].

### Retrieving moose HEV genome with high throughput sequencing

Triplicates of the liver sample positive for moose HEV RNA [28], were processed for the Illumina MiSeq platform sequencing as follows: The synthesized dsDNA was diluted and prepared with Qubit dsDNA HS assay kit (Life technologies, USA) according to manufacturer’s protocol and the concentration was measured with Qubit 2.0 Fluorometer (Life technologies, USA). A 1ng sample (0.2 ng/μl) was index library tagged with I5 and I7 primers and fragmented at the same time (tagmented) through a 5-cycle PCR amplification using the Illumina Nextera XT kit, according to Illumina MiSec protocol. The samples were loaded on a chip and analyzed on a Bioanalyzer (Agilent Genomics, Germany) for DNA concentration, size, and size distribution. The DNA samples were diluted, pooled and a total input of 1 ng DNA was loaded into a cartridge containing Technologies MiSeq v2 Reagent 300 cycle kit, according to MiSec protocol. The assembled contigs from reads generated through de novo assembly with default settings in the CLC Genomics Workbench 6.0 were BLAST-searched for HEV and all putative HEV contigs were subsequently assembled into several larger consensus sequences. All remaining non-related HEV consensus sequences were removed. The 5kb algSWE2013 (KF951328.1) sequence was compared with the MiSeq assembled HEV sequence, and the putative 5’-UTR terminal start position was identified using Gt1 (AY230202), Gt3 (EU360977) [31] and Gt4 HQ634346 genomes as templates. However, the Gt2 was not included because the 5’-UTR sequence was incomplete. Identification of putative HEV domains was done according to Koonin *et al*., 1992 [32] and the NCBI “ORF Finder” [33] was used for exploring putative ORFs.

### Statistical analyses

One sample Z-test for proportions was used to estimate the apparent prevalence confidence intervals (CIs). A χ^2^-test for equality of two proportions was used for analysis of significant difference (p<0.05) within moose age classes, sex and Swedish counties. All statistical analysis were performed in R, version 3.0.2.

### Phylogenetic analyses

The phylogenetic HEV relationships were determined with MEGA 5.0 [34] with maximum likelihood (ML) and neighbor-joining (NJ) approach using the Tamura-Nei evolutionary model. The first ML-tree was based on the shorter partial HEV RdRp sequence alignment with default settings. The less computational NJ approach was based on a codon alignment of concatenated complete HEV ORF1 and 2 with γ-value of 1.4. Bootstrap analysis with 1,000 replicas was used and values >70%, indicating statistical significant formation of clades, are indicated at the branches. The HEV sequences used here were twelve new partial moose HEV sequences (KP640874, KP640875, KP640876, KP640877, KP640878, KP640879, KP640880, KP640881, KP640882, KP640883, KP640884 and KP640885), and the following 26 sequences obtained from GenBank: KF951328.2 (updated moose HEV sequence), M80581, X98292, AY230202, AF051830, AY204877, M74506, FJ906895, FJ906896, EU360977, AB591734, AF082843, AB222182, AB189071, AB573435, GU119961, AB602441, GU954430, GU345042, GU345043, AY594199, AB521806, JQ001749, JN998606, HQ731015 and AM943646.

## Results

Pathological examination showed that no lesions were observed in any of the moose carcasses. Indication of past or ongoing HEV infection was found in 67 (29%) of 231 animals (Table 2). HEV RNA, indicating ongoing infection, was found in samples from 34 (15%) animals, but 24 of the 34 animals had no detectable anti-HEV antibodies. This marker of past HEV infection was found in 33 (14%) of the animals and 10 individuals (4%) had both detectable anti-HEV antibodies and HEV RNA (Table 2). The mean ct-value for the samples investigated by the qPCR was 34.5±4.1, (ct-range 25–38) indicating a low concentration of HEV RNA in most samples. Both serum and fecal samples were available for 51 animals. Four out of these were HEV RNA-positive in feces only, while an additional six samples were positive for HEV RNA in both sera and feces. Two of these latter animals were also seropositive for anti-HEV antibodies. HEV RNA was detected in 18 of 107 (16.8%) of the 0–1.5 year old moose, which was the highest proportion of active HEV infection for all age classes (Table 2), but the difference was not significant. However, the older moose (aged 2–4.5 years) had significantly higher prevalence of antibodies against HEV than the younger 0–1.5 year old (χ^2^ = 5.4 and p = 0.02), which indicates that HEV immunity increases with age. There was no significant difference in the total number of markers of present or past HEV infection between genders of moose (24/89, 27% males vs. 26/97, 27% females had HEV markers), although there was a larger proportion of ongoing HEV infection in males and markers of past infection in females (Table 2). The prevalence of these markers, HEV RNA and anti-HEV antibodies, did not differ significantly between moose from the counties in mid- and southern Sweden. However no marker was found in any of the 8 animals from the county Västerbotten, which is situated in the northern part of the country (Fig 1, Table 2).

**Table 2:** The number (N = 231) and proportion of moose positive for HEV RNA and/or anti-HEV antibody detected in serum, feces or liver, arranged according to age class, gender and Swedish county.

Variable	(N)	With anti-HEV (N)	(%, 95% Cl)	Reactive for HEV RNA (N)	(%, 95% Cl)	Total HEV markers* (N) (Both markers)	(%, 95% Cl)
**Age (years)**							
0–1.5	107	12	(11.2%; 6.0–19.2)	18	(16.8%, 10–25)	26 (4)	(26%, 18–35.2)
2–4.5	65	17	(26.2%, 16.0–38.5)	7	(10.8%, 4.4–20.9)	21 (3)	(32.3%, 21.2–45)
5.5–15.5	35	6	(17.4%, 6.6–33.7)	5	(14.3%, 4.8–30.3)	8 (3)	(22.9%, 10.4–40.1)
Unknown	24	8	(33.3%, 16.4–55.3)	4	(16.7%, 4.7–37.4)	12 (0)	(50%, 29.1–70.9)
**Gender**							
Male	89	14	(15.7%, 8.9–25)	14	(15.7%, 8.9–25)	24 (4)	(27%, 18.1–37.2)
Female	97	21	(21.6%, 13.9–31.2)	9	(9.3%, 4.3–16.9)	26 (4)	(26.8%, 18.3–36.8)
Unknown	45	8	(17.8%, 8–32)	11	(24.4%, 12.9–39.5)	17 (2)	(37.8%, 23.8–53.5)
**Swedish county**							
Öland	49	9	(18.4%, 9.2–32.5)	3	(6.1%, 1.6–17.9)	11 (1)	(22.4%, 12.2–37.0)
Småland	51	9	(17.6%, 8.9–31.4)	4	(7.8%, 2.5–19.7)	10 (3)	(19.6%, 10.3–33.5)
Västergötland	33	10	(30.3%, 16.2–48.9)	6	(18.2%, 7.6–36.1)	13 (3)	(39.4%, 23.4–57.8)
Södermanland	77	13	(16.9%, 9.6–27.5)	19	(24.7%, 15.9–36.0)	29 (3)	(37.7%, 27.1–49.5)
Västermanland	10	1	(10%, 5,2–45.9)	2	(20.0%, 0.30–55.8)	3 (0)	(30%, 8,1–64.6)
Värmland	3	1	(33.3%, 0.2–84.4)	0	(0, 0–0,7)	1 (0)	(33.3%, 18–87.7)
Västerbotten	8	0	(0%, 0–40.2)	0	(0,0–40.2)	0 (0)	(0,0–40.2)
**Total**	231**	**43**	**(18.6%, 13.9–24.4)**	34	**(14.7%, 10.5–20.1)**	**67 (10)**	**(29%, 23.3–35.4)**

* = 10 animals had both HEV-specific antibodies and HEV RNA detectable in their samples.

** = for 51 animals, both serum and fecal samples were available, 10 had detectable HEV RNA in feces, 6 of those had HEV RNA also in their serum samples.

**Fig 2:**
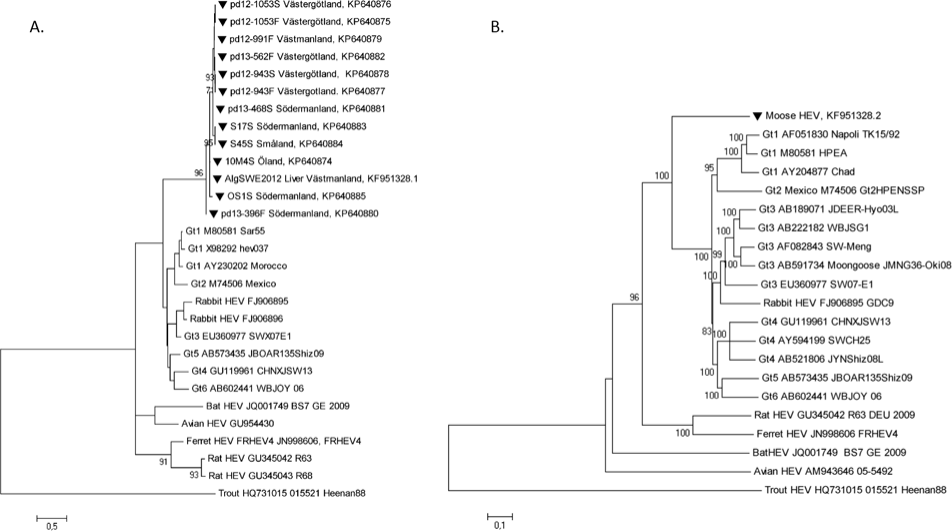
Phylogenetic relationship of moose HEV to other HEVs. Mega 5.0 was used to generate phylogenetic trees with 1,000 bootstrap replicas and bootstrap values >70% are indicated. **a)** Maximum likelihood tree based on the 324nt of the partial HEV RNA dependent RNA polymerase gene, avian and rat HEV were used as outgroups. Samples pd12-1053S (serum) and pd12-1053F (feces) were from the same moose, while pd12-943S (serum) and pd12-943F (feces) were taken from another moose. **b)** Neighbor joining tree based on ~7 kb nucleotides of in frame HEV ORF1 concatenated with ORF2 (representing full genome analysis); trout HEV was used as outgroup. The length of the bar indicates genetic distance. ▼: Moose HEV

Thirteen of the 34 HEV RNA-positive samples could be amplified by PCR and the partial HEV RdRp in ORF1 was sequenced. The phylogenetic relationship of this region revealed a high similarity (≥91%) between the strains, with mainly synonymous mutations and only one amino acid (aa) substitution, either as W or R at the N-terminal end. The phylogenetic analysis of these strains confirmed the formation of a distinct and separate moose HEV lineage, which shared a common ancestor with the Gt1-6 within the species *Orthohepevirus A* [3] or with the proposed genus *Orthohepevirus* [4] (Fig 2a). The moose strains formed no specific geographical clades based on origin, and sequences from feces and blood were identical for two animals (pd12-1053 and pd12-943). Serum samples S17S and 10M4S were amplified by nested PCR a second time due to of low concentration of amplicon after the first nested PCR.

The HEV strain AlgHEV2013 (KF951328.1) from a liver sample [28] was applied to the Illumina MiSeq platform for determination of the potential full genome sequence. The average fragment size ranged from 354 and 415bp after library tagmentation. By using BLAST, 32 contigs were identified, corresponding to 1,859 (0.014%) out of a total of about 13 million reads. The assembly of the putative moose HEV contigs resulted in a 6,976 nt long sequence (7,002 nt with the polyA tail), (Accession number KF951328.2) with an average sequencing depth of 37 across the near-complete HEV genome. All nt or aa position were referred to the KF951328.2 sequence, if nothing else was stated. The sequence had a 35–60% nt identity when compared to other HEV variants (Table 3), with the highest identity to Gt1-6: in the *Orthohepevirus A* species [3] or with the proposed genus *Orthohepevirus* [4]. It was 99% identical to the 5.1kb AlgHEV2013 sequence [28], with eight nt differences consisting in; four Y-nt ambiguities (4012, 4584, 4821 and 6138), and four synonymous substitutions (1927, 2536, 3808 and 6834). The putative moose HEV 5’-UTR terminal region was eight to fourteen nt longer than that in the Gt1 and Gt3-4 (S1 Fig). All three open reading frames (ORF1-ORF3) characteristic for HEV could also be identified (Table 3 and S2 Fig). The putative six regions; the methyltransferase, Y-domain, papain like protease (PCP), hypervariable region (HVR), X-domain, and RNA dependent RNA-polymerase (RdRp), observed in ORF1 in other HEV stains, could also be found in the complete ORF1 sequence of the moose HEV. The most divergent regions were found in the PCP, HVR (Table 3), and in ORF3 with 20–56% nucleotide, and 7–35% amino acid sequence identity to other hepeviruses [28]. The PCP and the HVR were 151 nt shorter and 41 nt longer, respectively, in the moose HEV compared to the Swedish Gt3 swine strain SWX07-E1 [31]. A concatenated moose HEV sequence of ORF1- and ORF2-based phylogenetic tree showed the same segregation as the partial moose HEV ORF1-based phylogenetic tree (Fig 2a and 2b). An additional twelve putative and potentially protein coding ORFs may exist throughout the moose HEV genome (S2 Fig).

**Table 3:** Nucleotide and amino acid sequence comparison of the moose HEV genome to other HEV genomes.

HEV Variant*	Full genome	ORF1	Methyltransferase	Y-domain	PCP	HVR
		38–4885 nt	203–748 nt	683–1312 nt	1334–1681 nt	1832–2215 nt
		1–1625 aa	56–237 aa	216–425 aa	433–548 aa	599–726 aa
	% identity / Length (nt)	% identity nt / aa	% identity nt / aa	% identity nt / aa	% identity nt / aa	% identity nt / aa
Gt1 (1)	59.5/7199	56.7/58.3	61.9/65.4	67.4/77.0	36.4/29.3	34.4/23.9
Gt2 (1)	59.1/7180	55.8/58.1	60.4/67.0	68.8/76.5	35.6/31.1	30.5/21.5
Gt3 (5)	59.7/7237	56.2/57.7	60.9/66.2	66.3/75.6	36.6/29.8	36.1/23.7
Rabbit (1)	58.2/7318	54.4/56.1	59.0/65.4	65.8/73.7	36.6/31.1	32.1/20.8
Gt4 (3)	59.1/7252	55.6/57.4	60.0/64.6	64.5/74.0	37.6/31.5	34.2/22.1
Gt5 (1)	59.0/7267	55.7/47.6	60.8/59.3	65.9/75.6	36.0/30.5	33.9/24.6
Gt6 (1)	59.2/7267	55.8/47.4	60.8/59.3	68.1/76.5	35.6/30.5	35.4/22.3
Rat (1)	50.2/6965	49.2/48.6	56.8/59.3	56.7/58.0	29.0/21.4	26.4/20.8
Ferret (1)	50.1/6854	49.4/48.7	56.6/58.2	54.1/57.2	30.5/20.6	23.1/16.4
Bat (1)	45.8/6796	47.0/42.2	54.8/56.3	50.1/50.2	28.0/13.6	23.3/13.2
Avian (1)	45.1/6631	46.5/41.7	52.8/53.0	54.4/52.3	26.7/15.7	21.8/14.8
Fish (1)	35.1/7310	36.3/24.2	40.0/25.8	37.3/23.4	23.0/8.9	28.0/10.8

The moose HEV ORF1 with some internally selected domains: methyltransferase, Y-domain, PCP and HVR were compared to other HEVs. Subsequent analyses of the helicase and RdRp of ORF1, 1968 nt ORF2 and 348 nt ORF3 were presented in a previous study [28].

*Compared hepevirus sequences obtained from Genbank, gt1:AY204877; gt2:M74506; gt3:AB222182, AF082843, AB591734, EU360977 and FJ906895; gt4:AY594199, GU119961 and AB521806; gt5:AB573435; gt6:AB602441; Rat HEV: GU345042; Ferret HEV: JN998606; Bat HEV: JQ001749; Avian HEV: AM943646; Fish HEV: HQ731015.

## Discussion

In this study it is shown that HEV infection is as common in moose as in wild boar in Sweden [12]. The zoonotic potential of this virus is not known but needs to be investigated since, as stated by WHO, about 75% of the new diseases that have affected humans over the past ten years have been caused by pathogens originating from an animal or from products of animal origin [35]. HEV is known to infect several animal species and is transmitted to humans by the fecal-oral route through contaminated food and water [8]. Interspecies HEV transmission, e.g. between wild animals like wild boar and deer, may also occur [20]. Such complex interspecies passages may contribute to geographic clustering of genetic similarities among porcine and human HEV isolates [12,36,37]. Different animals may thus be reservoirs for sustained zoonotic transmissions of HEV.

The detection of HEV RNA in feces (ten animals) indicated a potential fecal-oral transmission route between moose, and this transmission route has been observed in studies with other animal species e.g. with the domestic swine [8]. The occurrence of HEV RNA in both serum and feces (six animals), or in feces only (four animals), probably reflects different stages in the infection cycle. The virus shedding in feces appears to occur for a more extended time compared to the viremic phase in serum [38], as has been shown for domestic pig and humans [39,40]. Other transmission routes of HEV between the animals can however not be ruled out and need to be investigated. The role of the passive immunity through maternal antibodies against HEV in calves is still unexplored, but the calves should be potentially more susceptible to HEV infection after weaning at about 5–6 months of age. This could explain why several of the younger animals studied were viremic. It is still unknown how long they remain viremic, if immunity is life-long, if moose antibodies neutralize other HEV strains, or if the animal can be re-infected with HEV from moose or other HEVs. The majority of the moose samples were collected during autumn after the mating period. Moose reach puberty and start mating at the age of 1.5 years, during which time physical contacts between animals increase (e.g. fights and mating) and may be another factor in HEV transmission.

Pathological examinations of moose carcasses in this study showed that most of them were in good condition, suggesting that their HEV infection was mild or even subclinical, similar to HEV-infected swine [41,42]. However, more studies are needed to confirm this. Most of the moose in this study, 173 animals, had been previously screened for infection with tick-borne *Anaplasma phagocytophilum* [29]. All had markers of *Anaplasma*, and signs of ongoing infections peaked in the age 0–2 years [29], which was the similar age class for ongoing HEV infections. *Anaplasma*, is known to have immunosuppressive properties [43] and may increase the susceptibility of moose to HEV infection and prolong the infection time period, as has been seen in immunosuppressed humans [2]. It should not be ruled out that ticks also can constitute a vehicle for HEV transmission, however more studies are required to confirm this hypothesis.

No significant difference in incidence of past or ongoing HEV infection was found between the sexes, but there were differences among the different age groups, with the older animals having antibodies against HEV more often than younger animals, and a trend that younger animals more frequently had ongoing infections. A similar trend has been shown among wild boar, where ongoing HEV infection was more frequent in up to 2 year old animals [44]. Infection markers were found in moose from all regions of Sweden apart from those in the northern part of the country, indicating that there may be regional differences of HEV prevalence. The population density of moose varies on a national, regional, and local scale in Sweden, but the general trend (reflected in hunting bag statistics [45]), suggests that the density of moose in northern Sweden is lower than in the southern and middle parts of the country. These differences in moose densities may affect the transmission pattern, or it may be that the virus is recently introduced in the moose population and has not yet reached the northern parts of the country. More samples from different regions are needed to confirm this result. The sequences of the moose HEV strains were very similar to each other in the investigated RdRp region of ORF1, and formed a distinct lineage in the phylogenetic tree. It is not clear why only 13/34 (38%) of the HEV RNA-positive samples could only be partially RdRp sequenced. However, failure to sequence HEV appear to be common, for example in a study, where only 9/16 (56%) and 3/9 (33%) of qPCR HEV-positive samples from wild boar and red deer could be sequenced after nested-PCR amplification [46]. In another study it was demonstrated that conventional PCR assays targeting different regions of the HEV genome could only detect a proportion of HEV positive (as demonstrated by serology and qPCR) reference samples [47]. This could be due to genetic variability of the strains, thereby explaining why highly similar moose RdRp sequences showed a similar ability to be sequenced in this study. Repeatedly freeze and thawing of clinical samples- and extracted RNA-samples cause HEV RNA degradation and could thus explain the difficulties of amplifying HEV by PCR in some samples. To investigate if there are subgroups or putative genotypes of moose HEV, more samples from different geographical regions and comparison of several genomic regions or complete HEV genomes are needed. The lack of geographical clades raises questions concerning moose migration between the counties, whether there are different behavioral and transmission routes, or whether the virus was introduced recently into the moose population and therefore has not yet diverged into geographically distinct strains, as previously observed with Swedish Gt3 HEV isolates from domestic swine and human [12,36]. Conversely, the strains may also be more divergent than previously anticipated, which could explain the low success rate for RdRp sequencing of 38% may be due to an increased genetic variability. The ELISA assay used in this study could not discriminate between antibodies directed against different genotypes or variants of hepeviruses, which means that potential previous Gt3-4 infection could not be specifically identified in moose. However, a Gt1-4 HEV qPCR based assay [28], was performed in parallel with the moose HEV qPCR in this study and no Gt3-4-like HEV sequence was detected in the samples (data not shown). This may indicate a lower frequency or absence of infection with these genotypes in moose compared to what has been observed for Gt3-4 infections in other deer species [18,20,21,26], but a larger sample dataset is required to determine this. We know now that HEV infections are common in Swedish moose, but it would also be interesting to compare the HEV prevalence profile in free-ranging moose from other countries.

The generated HEV genome sequence from moose presented in this study had a 35–60% nt sequence identity compared to other hepeviruses. The shorter PCP and the extended HVR of moose HEV ORF1 were highly divergent and may play a role in the determination of host range. Additional putative ORFs were detected throughout the moose HEV genome, none of which were similar to the putative ORFs found at the 5’ terminal end in rat and ferret HEV genomes [14,17]. Conservation of these putative ORFs in other moose HEV genomes may indicate important biological functions e.g. encoding additional unknown viral proteins, however additional studies are required to confirm this. In the absence of an updated unified HEV classification system, two consensus classifications were recently proposed for members of the *Hepeviridae* family [3,4]. The distinct monophyletic moose HEV sequences shared a common ancestor with the sequences of Gt1-6 group, which indicates that the moose HEV would belong to the proposed *Orthohepevirus A* species, which includes Gt1-6 as members [3], or to the proposed genus *Orthohepevirus* [4].

In order to limit the spillover of zoonotic agents and protect human health, improved surveillance of wildlife pathogens is required in order to explore pathogen prevalence and the presence of factors for host specificity. This study showed a surprisingly high frequency of current and past HEV infection in moose and since moose meat is consumed on a regular basis in countries where moose is hunted, it cannot be ruled out that this species could be a potential reservoir for zoonotic transmissions. Unawareness of the requirements for proper hygienic handling and the consumption of undercooked moose meat may pose a risk for human HEV infections. The genetic similarity of moose HEV to the zoonotic Gt3-4 belonging to species *Orthohepevirus A* or to the proposed genus *Orthohepevirus*, may be indicative of a zoonotic potential also for the moose HEV. In addition, further studies are needed to investigate if moose can become infected by HEV Gt3-4, and if moose HEV can infect other species of deer, in order to better understand the host range and zoonotic properties of this virus.

## Supporting Information

S1 Fig The putative 5’-UTR of moose HEV, compared to the Gt1, Gt3 and Gt4. The only available published Gt2 HEV genome lacks the 5’-UTR and was not included here. The ORF1 start-codon is highlighted in grey and indicated by an arrow.
(TIF)

S2 Fig Location of moose HEV ORF1-3 and twelve putative ORFs A-M illustrated within the 6,976 nt of the moose HEV genome, which is 7,002 nt including the polyA tail. The putative positions of the coding regions have been obtained from the NCBI ORF finder tool.
(TIF)
