# Supplementary figures and images for: High Prevalence of Hepatitis E Virus in Swedish Moose – A Phylogenetic Characterization and Comparison of the Virus from Different Regions
Source: PLoS One. 2015 Apr 23;10(4):e0122102. doi: 10.1371/journal.pone.0122102 (PMC4408071; doi:10.1371/journal.pone.0122102)

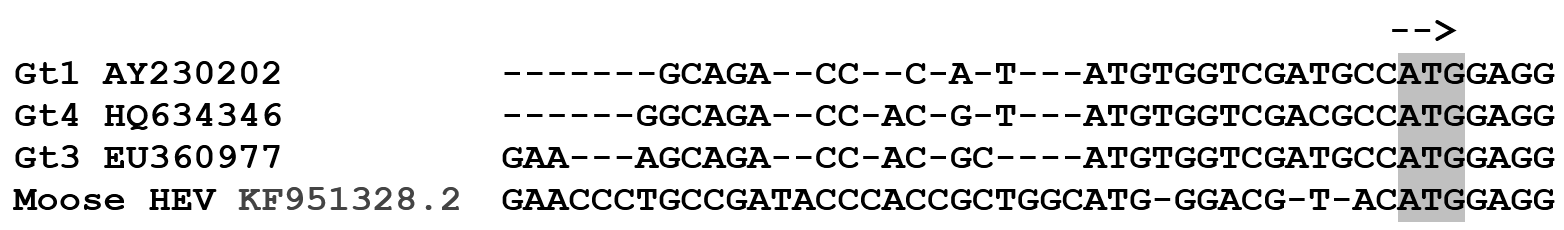

Supplement: S1 Fig — The only available published Gt2 HEV genome lacks the 5’-UTR and was not included here. The ORF1 start-codon is highlighted in grey and indicated by an arrow. (TIF) [file pone.0122102.s001.tif]

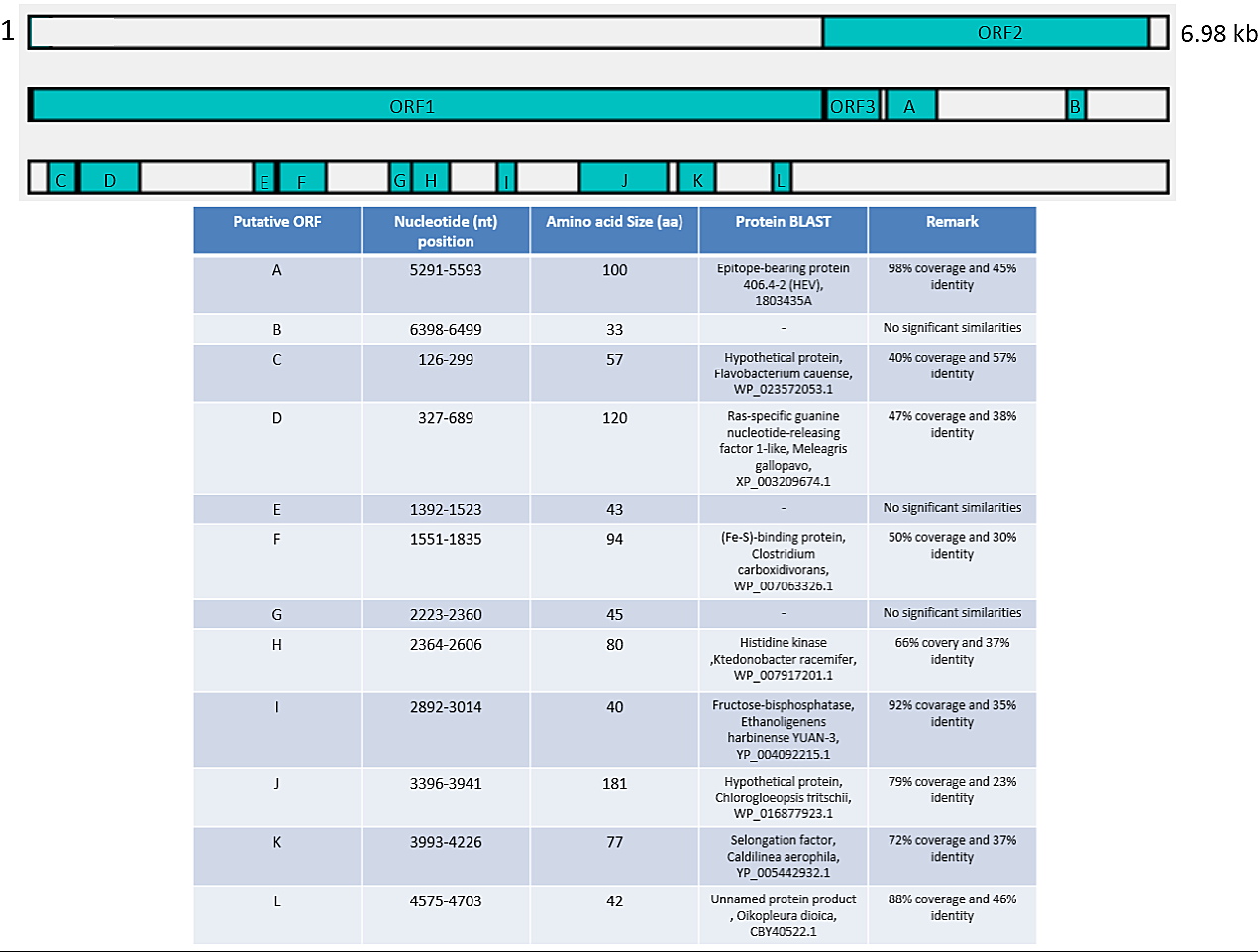

Supplement: S2 Fig — The putative positions of the coding regions have been obtained from the NCBI ORF finder tool. (TIF) [file pone.0122102.s002.tif]
